# Patient Perspectives on Prior Authorization for Cancer Care

**DOI:** 10.1001/jamanetworkopen.2025.23807

**Published:** 2025-07-29

**Authors:** Bridgette Thom, Sonia Persaud, Lauren V. Ghazal, Fumiko Chino

**Affiliations:** 1 University of North Carolina (UNC) School of Social Work, Chapel Hill; 2 UNC Lineberger Comprehensive Cancer Center, Chapel Hill, North Carolina; 3 Memorial Sloan Kettering Cancer Center, New York, New York; 4 University of Pittsburgh, Department of Health Policy and Management, Pittsburgh, Pennsylvania; 5 University of Rochester School of Nursing, Rochester, New York; 6 MD Anderson Cancer Center, Houston, Texas

## Abstract

**Importance:**

Health insurance companies may require prior authorization at any stage in the cancer care continuum, leading to the potential for denials and/or delays of diagnosis, treatment, or survivorship care. Patient experiences with prior authorization are largely unexplored, with most surveys focused on clinicians.

**Objective:**

To characterize prior authorization for cancer care from the perspective of patients with cancer, using their own words.

**Design, Setting, and Participants:**

This qualitative study provided a secondary thematic analysis of an online survey created in collaboration with patient advocates. Data were collected from July to October 2022. The survey was offered through a secure online platform. Participants included a convenience sample of patients with cancer, who self-reported being older than 18 years and having previous experience with prior authorization, recruited from social media, email lists, and word of mouth. Data were coded in May 2023, with initial themes constructed in September 2023.

**Main Outcome and Measures:**

Responses to the open-ended prompt were coded inductively by trained members of the research team, and themes were developed by the study team through discussion.

**Results:**

Of 178 respondents in the parent quantitative survey, 89 (50%) provided free-text responses. Most respondents to the open-ended question were women (79 [89%]) and aged 18 to 39 (35 [39%]) or 40 to 54 (31 [35%]). Codes from responses related to patient experiences, views of the process, and perception of the role of the health care team. Four interconnected themes were created from the codes: blinded navigation, intersecting burdens, interference with care, and a broken system.

**Conclusions and Relevance:**

In this qualitative secondary analysis of patient experiences with prior authorization, themes centered on how, from the patient perspective, prior authorization was a confusing process that added to the administrative, psychosocial, and financial burdens of cancer care. Intersecting burdens amplified the negative impact of prior authorization on patients. Patients voiced concerns that highlighted the broken, profit-driven nature of the United States health care system. Advocacy efforts are needed to promote reform to ensure patients receive timely access to recommended care, and policy considerations toward this reform must center the patient experience.

## Key Points

**Question:**

How do patients with cancer describe their experiences with prior authorization for cancer care?

**Findings:**

In this qualitative study of 89 participants, 4 interconnected themes emerged from the responses to an open-ended text prompt: blinded navigation, intersecting burdens, interference with care, and a broken system. Themes described patients’ perspectives that prior authorization is a confusing process that adds to the administrative, psychosocial, and financial burdens of cancer care.

**Meaning:**

These findings suggest that prior authorization processes can lead to negative outcomes for patients, and efforts toward health insurance reform must be inclusive of the patient voice.

## Teaser

This qualitative study characterizes the perspectives of patients with cancer regarding their experience with prior authorization for cancer care.

## Introduction

Health insurance companies have employed utilization management processes with increasing frequency, purportedly in order to reduce costs and limit unnecessary medical care.^1^ An often-applied utilization management process is prior authorization, in which clinicians must obtain payer approval before providing medical care for that care to be covered financially.^2^ Prior authorization can be required at any stage in the cancer care continuum, including for diagnostic imaging, anticancer treatment (ie, chemotherapy, immunotherapy, surgery, radiation therapy), genetic testing, supportive care, and survivorship care.^2^ As such, prior authorization can delay diagnosis, treatment, symptom management, and continuity of care and potentially be harmful to patients with cancer, given the medical acuity of their care needs.^3,4^

Emergent literature^5,6^ has characterized physician perspectives on prior authorization. In a survey of 394 oncology practices representing more than one-half of hematologists and oncologists in the US,^5^ prior authorization and denials and appeals were identified as significant pressures on their practices. A recent survey of radiation oncologists^6^ echoed these findings: due to prior authorization, 92% of 730 respondents reported treatment delays for patients; 33% reported abandoned treatments; and 30% said it led to adverse patient events.

Physicians have noted that patients are concerned about the impact of prior authorization on both their care and their financial status^7^; however, patient views of the process are largely unexplored. In prior work, Chino et al^8^ used survey methods to understand patient experiences of prior authorization, including patient involvement, length of subsequent delays, and association with trust in the health care team and insurance company. Using a thematic analysis approach, this analysis characterizes prior authorization experiences from the patient perspective, in their own words.

## Methods

### Study Process

This is a qualitative secondary analysis of an online survey that was created in collaboration with patient advocates to understand the patient perspective of prior authorization.^8^ A convenience sample of participants was recruited from July to October 2022 using social media, advocacy organization email lists, and word of mouth. Potential participants were screened through self-report to confirm they were 18 years or older and had an experience with prior authorization during their treatment for cancer. The Memorial Sloan Kettering Cancer Center Institutional Review Board deemed the primary survey to meet the criteria for 45 CFR 46 Exemption 2, and, as such, written consent for participation was waived. Participants were not compensated for their responses. Quantitative findings from the parent survey were published previously.^8^ Qualitative findings are presented in accordance with Standards for Reporting Qualitative Research (SRQR) guidelines.

Data for this analysis were drawn from responses to an open-ended prompt in the primary survey: “Please tell us anything else you would like to share about the process of prior authorization.” The specific prompt was not mandatory for survey completion. Responses to the question were characterized by length and content using measures of central tendency. Demographic characteristics (eg, race and ethnicity, educational attainment, income, gender) and clinical data (eg, diagnosis, stage, time since diagnosis) were self-reported as part of the parent survey to descriptively characterize the sample, although respondents could select “prefer not to respond” if warranted. Responses were categorized as African American or Black; American Indian or Alaska Native; Asian; Hispanic, Latino, or Latinx; Native Hawaiian or Other Pacific Islander; White; or another race. Respondents could select multiple categories. Gender was self-identified as man, woman, nonbinary, transgender woman, transgender man, another gender, or prefer not to respond.

### Data Analysis

Data were coded in May 2023, with themes initially developed through September 2023. Themes were revisited and revised in September 2024. Given the limited amount of research on this topic, an inductive, data-driven thematic analysis approach was applied to assess open-ended responses, in which 2 trained study team members (B.T. and S.P.) read and reviewed responses to jointly create a coding scheme using QDA Miner, version 6 (Provalis Research).^9^ Disagreement on initial codes was resolved through discussion. Codes included characterizations of patients’ emotional responses and administrative burdens; their views on process transparency and communication; general feelings about insurance companies and the health care system; and concerns about access and association with treatment. To organize the data, initial codes were inductively categorized into 3 broad groups that captured (1) patient report of their own feelings (17 codes focused on patient feelings); (2) patient perceptions of the prior authorization process itself (14 codes focused on process); and (3) the role of the health care team in facilitating a prior authorization (7 codes focused on the health care team role).

From the codes, the first author (B.T.) developed preliminary themes, which were presented to the senior author (F.C.) and subsequently refined. A patient advocate member of the team (L.V.G.) then reviewed codes and themes and provided feedback as a proxy for member checking, given the anonymous nature of the data. Themes were finalized by the study team through discussion and in drafting of the manuscript.

## Results

Of 178 respondents in the parent quantitative survey, 89 (50%) provided free-text responses, sharing what they believed was important about prior authorization. Most respondents to the open-ended question were women (79 [89%], compared with 7 men [8%], 1 nonbinary [1%], and 2 preferred not to answer [2%]) and aged 18 to 39 (35 [39%]) or 40 to 54 (31 [35%]) years, similar to the demographic composition of the parent survey. Two respondents (2%) identified as African American or Black; 2 (2%), American Indian or Alaska Native; 6 (7%), Asian; 1 (1%), Native Hawaiian or Other Pacific Islander; 80 (90%), White; 2 (2%), another race; and 1 (1%), prefer not to respond. Three respondents identified as Hispanic, Latino, or Latinx. Table 1 provides complete sample details. Open-ended responses ranged from 4 to 342 words (median, 41 words).

**Table 1.** Sample Demographic Characteristics

Characteristic	No. (%) of respondents (n = 89)
Age, y	
18-39	35 (39)
40-54	31 (35)
55-64	14 (16)
≥65	9 (10)
Gender	
Women	79 (89)
Men	7 (8)
Nonbinary	1 (1)
Prefer not to respond	2 (2)
Race^a^	
African American or Black	2 (2)
American Indian or Alaska Native	2 (2)
Asian	6 (7)
Native Hawaiian or Other Pacific Islander	1 (1)
White	80 (91)
Another race^b^	2 (2)
Prefer not to respond	1 (1)
Ethnicity	
Hispanic, Latino, or Latinx	3 (3)
Non-Hispanic	85 (96)
Prefer not to respond	1 (1)
Insurance type(s)^a^	
Private	68 (76)
Marketplace	9 (10)
Medicare	10 (11)
Medicare Advantage	3 (3)
Medicare supplement (Medigap)	3 (3)
Medicare Part D	2 (2)
Medicaid	5 (6)
Tricare	3 (3)
Highest level of educational attainment	
High school	2 (2)
Some college	10 (11)
Associate’s degree	5 (6)
Bachelor’s degree	30 (34)
Graduate or professional degree	42 (47)
Current household income	
<$20 000	4 (5)
$20 000-$39 999	5 (6)
$40 000-$59 999	9 (10)
$60 000-$99 999	16 (18)
≥$100 000	40 (45)
Prefer not to respond	15 (17)

^a^ Respondents could select more than 1 answer.

^b^ Indicates not specified.

Four interconnected themes were created from the initial codes: blinded navigation, intersecting burdens, interference with care, and a broken system. Although overlap exists among the themes in their effect on patients, each theme represents a distinct experience for patients in their views of prior authorization. Table 2 includes illustrative quotations from each theme. Quotations presented are unedited, except for redaction of insurance company names and clarifications of terms and abbreviations, which appear in brackets. Results below demonstrate how each of the code categorizations (patient feelings, prior authorization process, and health care team role) were represented within each theme.

**Table 2.** Participant Quotations by Theme

Theme	Illustrative quotation
Blinded navigation	It came out if nowhere. I had been on my nausea med over a year, and, all of a sudden, I’m the middle of a round of chemo, they refused to refill it, causing me to run out setting off my nausea in the middle of a round.
	I was blindsided with a denial. When I called my insurance company, the representative I spoke with didn’t care…. It was the most ridiculous conversation where I was made to feel uneducated in the prior authorization process with health insurance.
	Prior authorization is a process that is completely closed off for the patient. Major decisions are made by insurance, sometimes—in arbitrary manner, frequently—with incomplete information, always without patient’s access to the process. There is no way for a patient to speak to prior authorization department to sort anything out or advocate for herself.
Intersecting burdens	I wrote a letter including further citations and N[ational] C[omprehensive] C[ancer] N[etwork] guidelines. My oncologist appealed. I was finally granted access after being out of the drug for approximately 10 days. I am angry as I know the only reasons I got this was my ability to fight back and the willingness of my oncologist as well.
	I have learned insurance companies don’t always want to say yes to the expensive test/scans at first. They just create more work for patient and cancer team to prove why it is needed. It just should be a little easier on all parties involved.
	I did quite a bit of research/literature review myself for several different prior authorizations, including writing letters supporting why it was evidence based to approve the care suggested by my cancer team.
	Patients should not have to deal with this on top of the stress of a cancer diagnosis, but if patients don’t step in and advocate for themselves their treatment will likely fall through the cracks.
	Dealing with delayed treatment due to prior authorization made me have some of the most intense anxiety since being diagnosed with de novo Stage IV M[etastatic] B[reast] C[ancer]. It was horrible for both myself and my husband. He missed 2 days of work trying to get it figured out.
Interference with care	The process required I delay care every week, which kept adding up exponentially.
	If I had been able to get my genetic testing authorized sooner, I could have skipped a lumpectomy and re-excision and gone straight to mastectomy. One surgery, one time under anesthesia, instead of 3.
	I’ve had multiple experiences where a treatment required prior authorization and was subsequently denied after I received the treatment. I have received bills for thousands of dollars, which I then had to fight the insurance company about on the back end.
	My insurance was very reluctant to allow me to go out of network even though no one in network could do the surgery. The surgery that could be done in network was more invasive, worse outcome, and less quality of life, but insurance didn’t care because I could still “survive.”
	I was denied life-saving chemotherapy because my insurance company thought it was too expensive. I had to appeal multiple times. They also denied prior authorization for genetic testing. I had to pay out of pocket.
A broken system	The system is horribly broken and hurts the patient unnecessarily.
	The health care system needs to change. I always told myself the stress of dealing with insurance was worse than the cancer.
	Insurance companies shouldn’t have a say in the care cancer (really anyone) patients receive when directed by a medical doctor.
	It’s ridiculous. There’s no way some retired doctor who has never worked in oncology, let alone with very rare types of cancers, should have the ability to determine if new treatments are approved. If there are only a handful of doctors in the country who specialize in a particular cancer, they should be the authority on what is best for their patients
	I think prior auth[orization] is a joke. If I’m a stage 4 cancer patient with an entire team of doctors/nurses saying this is what’s best, how can an insurance company who doesn’t even know me or my case make a choice for me like that. It’s gross.
	[Redacted] insurance is a joke. They basically wrote me off as a dead man. I hope none of their family members ever have to experience what I have.
	The fact that my care would be denied during active cancer treatment, meaning my cancer diagnosis was not enough of a reason to approve cancer care, is unconscionable. It makes me feel incredulous and hopeless. What a useless, parasitic system American health care is.

Blinded navigation described the perception that patients felt uncertain and often unsupported during the prior authorization experience. Patient codes included feelings of surprise and confusion (eg, “I was blindsided” and “It came out of nowhere”). Process codes within this theme included patient perception of a lack of adequate communication prior to and during the prior authorization process and regarding the rationale for the prior authorization. Process codes also noted that obtaining preapproval for elements of their care was “stressful and overwhelming” and marked by a lack of transparency (“[N]o one from the insurance company could explain why or would allow my doctor or me to talk with the clinical provider who was denying my PET [positron emission tomography] scan”).

This lack of transparency was underscored by codes to characterize what patients perceived as the arbitrary nature of prior authorization. Patients described how prior authorization varied by state and insurance provider (“I moved states, and in the new state my maintenance treatment has been denied”) and how it was applied to treatments that did not previously require prior authorization (“I had been on my nausea med[ication] over a year, and all of a sudden, I’m the middle of a round of chemo[therapy], they refused to refill it”).

Communication difficulty was a key code in this theme. Several respondents noted that the insurance representative with whom they communicated was unable to make decisions regarding the prior authorization (“Insurance companies don’t give any power to the people who answer the phone”) and experienced inconsistency in information provided (“Everyone I talked to gave me a different explanation”).

Health care team codes included feelings of a lack of team effort to support patients through the process, apathy on the part of the clinic team, and, again, a lack of communication among all parties. Not all health care team codes in this theme were negative; however, as some respondents noted, they relied wholly or in part on the health care team to complete the prior authorization.

Intersecting burdens described the additional administrative and psychosocial burdens patients endured because of their prior authorization. For respondents, these burdens intersected to amplify the distress they already experienced from cancer diagnosis and treatment (“I always told myself the stress of dealing with insurance was worse than the cancer”). Patient codes focused on feelings of anxiety and stress relating to the uncertainty prior authorization caused, both in terms of when it would be required and how it would impact care (“Also the fear that they may one day say no sen[t] [my] anxiety through the roof”).

Process codes described the administrative burden prior authorization caused, with several respondents noting the details of their engagement in the process, including making telephone calls, writing letters, creating social media campaigns, and conducting literature searches (Table 2). Process codes also included financial burdens that arose because of prior authorization (“I decided to pay out of pocket” and “I had to pay out of pocket”).

Respondents noted how communication difficulties with the health care team added to their administrative burden (“…the PCP [primary care physician] office never followed up with me like they said they would.…It was like this wasn’t important for them. I had to stay [on] top of everything” and “I often find that they forget to get authorizations, so I have to call in advance myself to insurance and then my doctor office”).

Interference with care referred to the outcomes associated with the prior authorization, both related to delays to diagnosis and treatment and to denials of recommended care. It also encapsulated the additional adverse effects, both physical and psychosocial, and the alterations in care plans that patients endured because of prior authorization.

Patient codes noted the impact of changes to the original care plan (eg, experiencing delays, having to take different medications) or the ways in which treatment had to be altered. For example, 1 patient described having to self-inject medications rather than receiving them from a clinician (“The process required I delay care every week, which kept adding up exponentially. It meant I had to give myself injections instead of receiving them from care providers”). Another patient, however, experienced the opposite and was no longer permitted by the insurance company to receive a treatment at home, as ordered, requiring trips into the cancer center for care: “When we found out [home administration] was a problem, I just went to the cancer center for the injections. It was frustrating….”

Similarly, other patients described having to alter the care plan because of a denial and outlined the impact of those alterations, which included worsening or additional symptoms in some cases: “…honestly, I was so tired and drained at that point that I just went with the ‘less effective’ care suggested” and “I had to take another med[ication] with horrible side effects rather than what my onc[ologist] prescribed. After I developed fever and body pain, they would let me change to the one originally prescribed.”

Patient codes in this theme also related to coping mechanisms and resultant outcomes patients described to manage denials of, limits to, or delays in care. For example, prior authorization caused 1 patient to ration a medication that was ultimately covered, but limited in quantity, by insurance: “I hoard it always afraid I am going to run out. I question myself, is the nausea that bad right now to take it? Should I just hold off or try another method? This has resulted in discomfort, distress, long days filled with nausea, and feeling hopeless.” Another patient described cutting pills in half to make them last longer when prior authorization caused a refill to be delayed: “I started cutting [the pain medication pills] in half to make them last longer (which wasn’t sufficiently addressing the pain) and then had to go a couple nights with only OTC [over-the-counter] meds.”

Process codes described how the steps of prior authorization, including peer-to-peer review, led to delays in care. For example, “The peer-to-peer appeal process took time away from patient care, and it showed that the person on the insurance side denying was not an oncologist or an expert in my treatment plan” and “The most frustrating prior authorization process took days and multiple professionals to complete for something the insurance cannot legally say no to.”

Relating to the health care team, respondents identified the need for teams to begin the process early to mitigate potential delays and denials (“Cancer teams need to get authorization well in advance of the time my oncologist wants scans/MRIs [magnetic resonance imaging]”). Respondents also highlighted the importance of hospital-based support programs for patients who face high out-of-pocket expenses because of insurance denials: “I wish my cancer center would have had better resources to address programs I could use to pay for the chemo my insurance denied. I was able to find the program but not everyone can.”

A broken system was an overarching theme that captured patient frustration with the prior authorization process. Patient codes within this theme highlighted the strong emotional response prior authorization elicited from respondents, including feelings of anger, despair, desperation, and disgust (Table 2).

Process codes focused on the profit-seeking nature of the insurance industry and the perception of respondents that insurance companies prioritize financial interest over their health (“It really made me feel like stage IV in the mind of [redacted insurance company] was a death sentence and we were not worth the hit to their bottom line”; “Insurance companies trying to save money is going to cost patients precious time and appropriate care”; and “It is largely part of the business plan of insurers to increase their profits and has little to do with patient well-being”).

Health care team codes in this theme broadly spoke to the trust respondents reported having in their care team and their beliefs that the clinician has the patient’s best interest in mind. This led to perceptions of unjustified interference in care on the part of the insurance company (eg, “I don’t understand how an insurance co[mpany] can decide my treatment by not [authorizing] certain tests. My doctor should be the only one decided which tests and treatments [I] get” and “Insurance companies need to stay out of patient care and leave that to the experts who know their patients and are best in the position to work with those very patients to decide their care and treatment plans”).

## Discussion

In this qualitative analysis, our findings highlight the patient perspective of prior authorization for cancer care. To patients, prior authorization is a confusing, opaque process that adds to the administrative, psychosocial, and financial burdens of cancer care. The intersection of these burdens amplifies the physical, psychological, and monetary impact on patients, who must also comanage the symptoms of their disease and the adverse effects of their cancer treatments. In addition, prior authorization can lead to negative downstream impacts on both care received and patient perception of care quality.

Nearly one-third of 730 surveyed oncologists noted that prior authorization led to an adverse event (eg, emergency room visit, hospitalization, permanent disability), and 7% stated it contributed to the death of one of their patients.^6^ Findings from our analysis support both the limited prior research in this space and prior analyses demonstrating the harmful impact on treatment adherence and outcomes.^8,10,11^ The challenges patients experience navigating a complex health system while facing a critical diagnosis have also been highlighted in recent literature outside of oncology, including patients within the Veterans Administration and those with rare and potentially fatal genetic conditions.^12,13^

Our findings echo the ongoing and growing dissatisfaction with for-profit health insurance companies recently amplified in mainstream media, demonstrating that patients have reached a tipping point in their pain and frustration with prior authorization and other forms of health management utilization.^14,15,16^ The widespread outrage about prior authorization has led to independent reporting on lucrative third-party contracts to reduce spending on medical procedures, with one company promising a 3-to-1 return on investment.^17^ This is mirrored in our findings that patients feel that their lives are being weighed against company profits.

In addition to the patient harm demonstrated in our findings, prior authorization can amplify growing disparities in access and outcomes among patients who have been historically and currently minoritized, marginalized, or excluded.^18^ As one respondent noted, “I am in medicine and know how to navigate the system and these issues make me cry and get frustrated very often, I can’t imagine how an elderly person or how someone who isn’t fluent in English gets by.” Navigating prior authorization requires technological savvy, an understanding of insurance processes, knowledge of personal rights, and time to engage in the process. Disparities in health insurance literacy disproportionately affect younger patients, those with lower educational attainment, and patients from minoritized racial and ethnic groups, with lower health insurance literacy being associated with more delayed or foregone care.^19,20,21,22^ Additionally, studies on the “time toxicity” of cancer care have demonstrated that the prior authorization process for patients and clinicians takes vital time away from completing other life responsibilities.^23,24^

### Limitations

Although our findings are novel in characterizing patients’ experience with prior authorization in their own words, there are several limitations to consider when interpreting the results. The responses are drawn from a convenience sample of respondents recruited from the internet and word-of-mouth, suggesting a highly engaged, self-activated sample with a particular interest in the topic. This sample is likely not representative of the broader US population of patients with cancer who require prior authorization for their cancer care; it is possible that findings underestimate or overestimate the impact of prior authorization on patients. Women, patients younger than 65 years, respondents with a college degree or higher, and non-Hispanic White patients were overrepresented in the sample relative to the US population of patients with cancer, which limits the generalizability of the results and can introduce biases. Last, despite our efforts to apply rigorous qualitative methods through team coding and theme building, these data are not representative of prospectively designed qualitative inquiry, as they are drawn from an open-ended text response. Nonetheless, these findings are important in their focus on patient experiences and provide a useful base from which to conduct further inquiry in broader, more diverse samples to better understand the impact of prior authorization by patient demographic and clinical characteristics and treatment modalities.

## Conclusions

This qualitative analysis highlights that intersecting burdens caused by the prior authorization process for cancer care amplify the negative impact of a cancer diagnosis and treatment on patients. Advocacy efforts are needed to promote prior authorization reform to ensure patients receive timely access to recommended care, and policy considerations toward reform must continue to center the patient experience.

## Notes

Supplement.
Data Sharing Statement
