# Supplementary material for: Patient Perspectives on Prior Authorization for Cancer Care
Source: JAMA Netw Open. 2025 Jul 29;8(7):e2523807. doi: 10.1001/jamanetworkopen.2025.23807 (PMC12308430; doi:10.1001/jamanetworkopen.2025.23807)
Supplement: Supplement. — Data Sharing Statement [file jamanetwopen-e2523807-s001.pdf]

Data Sharing Statement

Thom. Patient Perspectives on Prior Authorization for Cancer Care. *JAMA Netw Open*.
Published July 29, 2025. doi:10.1001/jamanetworkopen.2025.23807

Data
Data available: No

Additional Information
Explanation for why data not available: De-identified data will only be made available with a signed data use agreement and explicit permission from the IRB.
